# 

**Published:** 1859-07

**Authors:** 


*Dental Surgery.*—Mr. Tomes' System of Dental Surgery, announced several years ago, as in press, has recently been published by Mr. Churchill, London. It is a 16mo. volume of near six hundred pages. It is illustrated by well executed wood cuts. Two hundred and fifty-six pages are devoted to teething; forty-eight to the dental tissues; one hundred and twenty-four to caries, and the remainder of the book to other pathological conditions of the teeth. With regard to the merits of the work as a practical treatise, we are not able to speak very positively at this time, as we have only glanced rapidly over one or two hundred pages, but so far as we can form an opinion from what we have read, it seems to be very deficient in practical detail, and the style is somewhat affected. In our next number we may notice the work more fully.

